# Challenges in Diagnosis and Treatment of Pancreatic Exocrine Insufficiency among Patients with Pancreatic Ductal Adenocarcinoma

**DOI:** 10.3390/cancers15041331

**Published:** 2023-02-20

**Authors:** Xiaoyang Lan, Gabrielle Robin, Jessica Kasnik, Grace Wong, Omar Abdel-Rahman

**Affiliations:** 1 Department of Medicine, University of Alberta, Edmonton, AB T6G 2R3, Canada; 2 Nutrition Services, Cross Cancer Institute, Edmonton, AB T6G 1Z2, Canada; 3 Pharmacy Department, Cross Cancer Institute, Edmonton, AB T6G 1Z2, Canada; 4 Department of Oncology, Cross Cancer Institute, University of Alberta, Edmonton, AB T6G 1Z2, Canada

**Keywords:** PDAC, PEI, survival, prognosis, supportive care

## Abstract

**Simple Summary:**

Pancreatic ductal carcinomas (PDACs) are difficult to diagnose at an early stage and carry a poor overall prognosis for patients. Many patients with PDACs have serious nutritional deficiencies relating to their cancer. Typically, the pancreas produces enzymes resulting in food digestion. However, patients with pancreatic cancer often have a limited ability to digest their food due to decreased enzyme function, known as pancreatic enzyme insufficiency (PEI). These patients then become malnourished, which lowers their quality of life and reduces their survival. Unfortunately, PEI in PDACs is sometimes missed by healthcare providers. This paper reviews past studies describing malnutrition in PDAC, focusing on PEI and its treatment. It focuses on guidelines and recommendations for the appropriate treatment of PEI. We also review knowledge gaps of healthcare professionals regarding PEI to enhance treatment uptake and improve future patients’ quality of life.

**Abstract:**

Pancreatic ductal adenocarcinoma (PDAC) is the most common malignancy of the pancreas and is associated with an extremely poor prognosis. Many PDAC patients suffer from profound nutritional complications such as nutrient deficiencies, weight loss, malnutrition, and cancer cachexia. These complications cause barriers to effective anticancer treatments, gravely influence their quality of life, and decrease their overall survival. Pancreatic exocrine insufficiency (PEI) is defined as impaired digestion due to inadequate secretion of pancreatic enzymes and is a common cause of malnutrition in PDAC. This review first summarizes the existing literature around malnutrition in PDAC, with a particular focus on PEI and its management with pancreatic enzyme replacement therapy (PERT). Second, we summarize existing guidelines and recommendations for the management of PEI among patients with PDAC. Lastly, we highlight potential gaps of knowledge of PEI among healthcare providers resulting in underdiagnosis and treatment, which may have implications for the quality of life and overall survival of PDAC patients.

## 1. Introduction

Pancreatic ductal adenocarcinomas (PDACs) account for 80–90% of pancreatic cancers [1,2,3,4,5]. Arising primarily from pancreatic ductal cells, this malignancy carries the worst prognosis between pancreatic cancer subtypes [1,2,3,4,5,6]. Although there has been modest progress in the detection and management of pancreatic cancers, the overall prognosis remains poor [2,5]. A 2023 report from the American Cancer Society estimates that the 5-year overall survival of pancreatic cancer patients is approximately 12% [2,7]. Pancreatic cancers are currently the fourth leading cause of cancer-related death, and they are predicted to further increase in prevalence over the next several years [1,2]. 

The incidence of pancreatic cancer is correlated with age, where 80–90% of cases are diagnosed over the age of 55 [1,2,3]. Men also carry a higher incidence of pancreatic cancer than women (5.5 vs. 4.0 in 100,000) [1,2,5,8]. Geographically, pancreatic cancer is more common in developed regions such as North America and Western Europe [3,8,9]. This distribution is attributed to increased imaging and data access, as well as increased risk factor exposures [2,4,9].

Prominent risk factors for pancreatic cancer include smoking and family history [1,2,3]. Smoking is the most important environmental risk factor, increasing the risk of pancreatic cancer nearly two-fold in smokers when compared to non-smokers [1,4,5]. In Canada, approximately 17% of pancreatic cancers are attributed to smoking [2]. Family history is also pertinent, as 5–10% of patients with pancreatic cancer describe an associated pertinent family history [1,3,5]. Hereditary pancreatitis, Lynch syndrome, and certain genetic mutations, such as BRCA 1/2 and ATM, are implicated [2,4,5]. Some evidence exists for serial pancreatic cancer screening in high-risk populations such as those with a genetic predisposition [2,4,5]. Additional non-modifiable risk factors include ethnicity (highest in African Americans), diabetes mellitus (type I and II), chronic pancreatitis, and intraductal papillary mucinous neoplasms (IPMNs) [1,3,4,5]. Modifiable risk factors include excess alcohol intake (≥3 drinks per day), increased body weight, and the consumption of processed or fatty foods [1,2,3,4,5,8].

Based on the location of the pancreas in the abdomen, new pancreatic masses can develop for weeks to months without causing clinical signs or symptoms [2]. As such, early-stage pancreatic cancers are often missed as patients are typically asymptomatic [1,10]. Eventually, as the mass affects nearby structures, signs or symptoms begin to occur [2,6,10,11]. With late symptom onset, most (>60%) of pancreatic cancers are diagnosed at an advanced stage [1,2,11]. The most common clinical presentations include constitutional symptoms, pain, and jaundice [11,12], but may also include fatigue, anorexia with early satiety, dyspepsia, nausea, weight loss, pruritis, and acholic stool [1,2,3]. 

Typical late-stage diagnoses limit successful surgical resections, with ~80–85% being unresectable at the time of diagnosis [1,2,5,6,8,11,13]. However, surgical resection is the mainstay curative-intent treatment [2,5,8,11]. Importantly, prognostic factors such as TMN stage at diagnosis impact the feasibility of curative-intent surgical resection [1,4,12,13]. If patients proceed with surgery, adjuvant chemotherapy has been shown to delay recurrence and improve survival. First-line options for adjuvant treatment include combination therapy with modified FOLFIRINOX (oxaliplatin, irinotecan, leucovorin, and fluorouracil), Gemcitabine-Capecitabine combination [14], or single-agent gemcitabine [2,15]. Neoadjuvant chemotherapy has the potential to improve resection rates for locally advanced PDAC and is also an area of active investigation for resectable PDAC [16]. Of note, a recent meta-analysis of resectable PDAC demonstrated improved rates of R0 resection and overall survival with neoadjuvant chemotherapy [17]. 

Current systemic therapy options in the metastatic setting include modified FOLFIRINOX or gemcitabine +/− nab-paclitaxel [15,18,19]. Ultimately, palliative care consultation is recommended to ensure symptom management and psychosocial support are accessible to patients [2,5]. Earlier access to palliative and supportive treatments has been shown to improve outcomes among patients with advanced PDAC [20,21,22,23]. The spectrum of supportive care involvement in these cases includes (but is not limited to) pain management, psychosocial interventions, and nutritional interventions. 

## 2. Malnutrition and Pancreatic Cancer

Nutritional status in pancreatic cancer is negatively impacted through a variety of mechanisms and may have profound implications for patients’ quality of life as well as overall outcomes [24]. The diagnostic criteria for malnutrition in adults were defined in a consensus statement from the Academy of Nutrition and Dietetics and American Society for Parenteral and Enteral Nutrition, requiring two or more of the following: insufficient energy intake, weight loss, loss of muscle mass, loss of subcutaneous fat, localized or generalized fluid accumulation, or diminished hand-grip strength [25]. Nutritional status may also be assessed using standardized patient questionnaires, such as the Patient-Generated Subjective Global Assessment (PG-SGA) [26]. A PG-SGA score of 2 or higher indicates that pharmacological intervention is necessary for a patient’s nutrition [27]. Weight loss is also a well-recognized complication of pancreatic cancer and can often be present at the time of diagnosis as one of the earliest symptoms [24,28,29]. There are three proposed categories of weight loss in pancreatic cancer based on etiology: anorexia, malabsorption, and cachexia or sarcopenia [29]. Importantly, both weight loss and malnutrition have been demonstrated to negatively impact performance status, quality of life, and overall prognosis of pancreatic cancer patients [27,30]. 

Cancer cachexia is a syndrome of loss of skeletal muscle mass resulting from dysregulated metabolism and poor nutrition. In 2011, cancer cachexia was defined through international consensus as weight loss greater than 5% over 6 months, weight loss greater than 2% in individuals with body mass index (BMI) <20 kg/m^2^ or the presence of sarcopenia [31]. There also exists significant heterogeneity in cachexic patients, as it may manifest as three different clinical subtypes, including patients with fat and muscle wasting, fat-only wasting, and no wasting [32]. Approximately two-thirds of PDAC patients meet the criteria for cancer cachexia at the time of diagnosis [33]. The cause of cancer cachexia is multifactorial, driven by mechanical complications of cancer that impair food intake and absorption, as well as the release of tumor- and host-derived factors that result in systemic inflammation and catabolism [34]. Sarcopenia is defined by the International Working Group on Sarcopenia (IWGS) to include low skeletal muscle mass and either low muscle strength or low muscle performance [35].

Importantly, the presence of cachexia and/or sarcopenia may be associated with poorer outcomes in pancreatic cancer [36,37,38,39]. In a retrospective study of 408 patients who underwent pancreaticoduodenectomy for primary pancreatic adenocarcinoma, Pausch et al. [36] demonstrated a higher 90-day mortality for patients with BMI <18.5 kg/m^2^ and an increased risk of both operative and non-operative complications in patients suffering from unintentional weight loss. A 2015 systematic review from Ozola Zalite et al. [38] also concluded that severe weight loss and sarcopenic obesity may be negative prognostic factors for PDAC, although the analysis was limited by heterogeneity in the definition of cachexia. In a 2022 study, Hou et al. [40] further assessed the independent contributions of cancer cachexia and sarcopenia in 232 patients with advanced pancreatic cancer. In this cohort, the prevalence of cancer cachexia and sarcopenia was 83.6% and 49.1%, respectively [40]. Interestingly, while cancer cachexia was identified as a poor prognostic factor for all patients, sarcopenia was only a poor prognostic indicator for patients with a high BMI or those who were on chemotherapy [40]. Cachexia and sarcopenia are therefore partially overlapping clinical features for PDAC patients, with individual implications for patient outcomes [40].

Malnutrition can be a significant contributor to the functional decline of pancreatic cancer patients, with potential implications for whether patients are candidates for systemic treatment. FOLFIRINOX and Gemcitabine with nab-paclitaxel are the mainstays of palliative intent chemotherapy for PDAC [18,41]. However, the FOLFIRINOX study excluded patients with an Eastern Cooperative Oncology Group (ECOG) score of 2 or higher, while only 8% of patients in the study of Gemcitabine and nab-paclitaxel had an ECOG score of 2 [18,41]. Systemic treatment for patients with ECOG 2 in the metastatic setting is therefore limited to gemcitabine alone with or without nab-paclitaxel [42]. In a 2019 study, Bicakli et al. [27] demonstrated that worsening malnutrition as defined by the PG-SGA score is associated with a higher ECOG score and poorer survival outcomes. Strikingly, 100% of patients with an ECOG score of 2 or higher had a PG-SGA score between 3 to 4 [27]. These results are supported in a 2021 study by Santos et al. of 41 patients with pancreatic cancer that demonstrated a significant correlation between nutritional status and overall function [43]. 

Nutritional interventions, including dietitian consultation and the use of nutritional supplements for pancreatic cancer patients being treated with chemotherapy can improve quality of life and functional status [44,45]. The use of home parenteral intervention has also been demonstrated to result in improved quality of life, performance, and nutritional status [46,47]. Importantly, the use of parenteral nutrition to maintain patients’ nutritional and functional status can ensure longer treatment courses with potential impacts on survival [46]. Several pharmacological approaches have been trialed to target cancer cachexia in pancreatic cancer, including the use of progestogens, corticosteroids, cannabinoids, anti-inflammatory agents, and Omega-3 fatty acids [34,48]. There has also been increasing interest in the use of ghrelin receptor agonists in the targeted management of cancer cachexia [49,50]. For example, Hamauchi et al. [51] performed a multicenter, single-arm trial of the ghrelin receptor agonist Anamorelin in 50 patients with advanced and unresectable gastrointestinal cancers, demonstrating improvements in patient-reported appetite, nutritional status, as well as mean lean body mass (1.89 ± 0.36 kg) and body weight (1.41 ± 0.61 kg). 

In addition to specific nutritional interventions, systemic therapy for PDAC may also modify nutritional outcomes by directly targeting the underlying etiology of cachexia. In a 2018 study of 977 patients with PDAC, Hendifar et al. [33] demonstrated that cachectic patients not receiving treatment had a 40% increase in the risk of death, while cachectic patients receiving treatment had a similar prognosis to non-cachectic patients. Sandini et al. [52] examined the body composition of 193 pancreatic cancer patients who underwent neoadjuvant chemotherapy followed by surgical resection, demonstrating that patients experienced a loss of adipose tissue without a loss of lean mass. In addition, patients who were ultimately candidates for surgical resection experienced an average of 5.9% increase in skeletal muscle mass, compared with 1.7% decrease in those who were not surgical candidates [52]. This suggests that cancer directed treatments can downregulate tumor-associated signaling pathways that are responsible for the catabolic state in pancreatic cancer [52]. Overall, multiple lines of evidence demonstrate the impact of malnutrition on the functional status and outcomes of PDAC patients as well as highlight the importance of nutritional intervention as a standard component of cancer care. 

## 3. Diagnosis of Pancreatic Exocrine Insufficiency (PEI) in PDAC

Pancreatic exocrine functions are commonly affected in PDACs, causing many patients to be malnourished (30%), lose weight (80%), and become cachectic through a variety of mechanisms [6,13]. PEI is defined as a reduction in the activity of pancreatic enzymes within the gastrointestinal system that results in impaired digestion [53,54,55]. The most common etiology of PEI is chronic pancreatitis, although it may also be seen in conditions such as cystic fibrosis, type 1 and type 2 diabetes, celiac disease, inflammatory bowel disease, pancreatic cancer, and following pancreas surgery [54,55,56]. PEI may result from decreased production of pancreatic enzymes due to the loss of pancreatic tissue from cancer itself or surgical resection, obstruction of pancreatic ducts, decreased stimulation of pancreatic enzyme production, or asynchrony between secretion of pancreatic enzymes and meals [54,57]. Historical evidence suggests that the exocrine pancreas has a large functional reserve, and patients can remain asymptomatic until pancreatic lipase reaches 10% of normal levels [58]. Therefore, timely diagnosis of PEI requires a high degree of clinical suspicion and a standardized approach to both patient assessment and laboratory testing.

The symptoms of PEI are non-specific and can include gastrointestinal manifestations of steatorrhea, abdominal pain, bloating, flatulence, foul-smelling stools, diarrhea, and unexplained weight loss [54,59]. PEI may cause malabsorption in all macronutrients including fat, protein, complex carbohydrates, micronutrients, fat-soluble vitamins (Vitamins A, D, E, K), and lipoproteins [57]. Secondary complications such as reduced bone mineral density or osteoporosis due to vitamin D deficiency may occur [54]. In pancreatic cancer, PEI is a major contributor to weight loss and cachexia along with contributions from the systemic inflammatory response and tumor metabolism [60]. The severity of PEI may be classified into mild (reduced secretion of enzymes, with normal bicarbonate and fecal fat excretion), moderate (reduced enzyme secretion and bicarbonate with normal fecal fat excretion), or severe (reduced enzyme secretion, reduced bicarbonate concentration, and steatorrhea) [61,62]. Standardized screening tools for PEI such as the pancreatic exocrine insufficiency questionnaire (PEI-Q) have been developed, which may improve the accuracy of PEI diagnosis [63]. 

**Figure 1:**
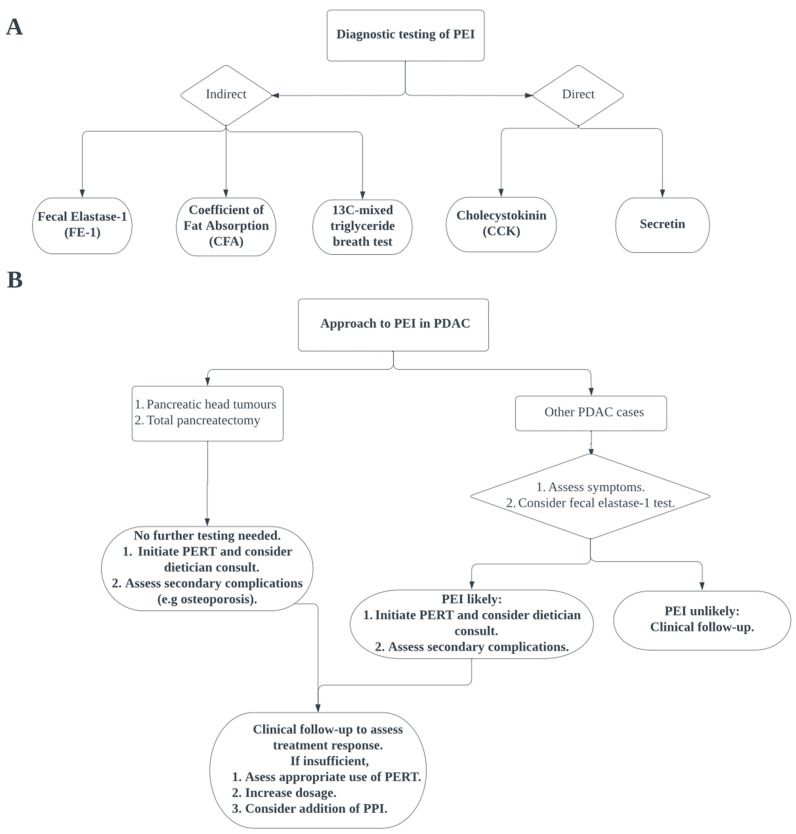
Suggested diagnostic testing (**A**) and approach (**B**) to pancreatic exocrine insufficiency (PEI) among patients with pancreatic ductal adenocarcinoma (PDAC).

There are multiple diagnostic tests available for PEI, both direct and indirect (Figure 1A). One indirect approach is the measurement of fecal elastase-1 (FE-1). FE-1 is an enzyme exclusively produced by the pancreas that is not susceptible to breakdown in the intestine, making it a measurement of the secretory capacity of the pancreas [55,60]. In pancreatic cancer, a severe reduction of FE-1 level (<20 mcg/g) is an independent predictor of poor survival [64]. Notably, the measurement of FE-1 is a reliable and reproducible screening tool for severe PEI when compared to secretin tests [65]. In a systematic review and meta-analysis, Vanga et al. [66] calculated an overall sensitivity of 0.77 and a specificity of 0.88 of the FE-1 assay in detecting PEI. They also identified an FE-1 level of above 200 mcg/g as a reasonable cut-off point to rule out PEI. However, FE-1 is not sensitive to mild to moderate PEI, where less than 50% of patients will have an abnormal FE-1 test [62]. 

Several additional indirect diagnostic tests for PEI have been described in the literature. First, the N-benzoyl-L-tyrosyl-p-aminobenzoic acid (BT-PABA) test measures the activity of chymotrypsin to identify exocrine insufficiency [67]. Second, the measurement of the coefficient of fat absorption (CFA) is currently considered the gold standard test for fat maldigestion. However, the CFA is not specific to PEI, requires adherence to a specific diet and collection of stool samples over 72 h, and is therefore challenging to obtain for clinical applications [57,68]. Lastly, Dominguez-Munoz et al. [69] developed a non-invasive breath test which measures the breakdown product of a standard ^13^C-labeled fatty substrate. The ^13^C-mixed triglyceride breath test is well correlated to CFA and is less cumbersome to perform [69]. However, this test is less sensitive to mild PEI and is not widely available in clinical settings [55]. Presently, no laboratory testing beyond the FE-1 is widely available for routine clinical use for PDAC patients. 

Direct approaches to the measurement of PEI are generally more sensitive and involve measuring pancreatic secretion following stimulation with either cholecystokinin (CCK) or secretin. Traditionally, this requires intubation of the duodenum. An alternative approach that uses sample collection using upper endoscopy may not be sufficiently sensitive and specific for routine clinical application [70,71]. Regardless, direct testing of PEI is labor-intensive and more invasive, and therefore may not be appropriate for the pancreatic cancer population. As no single diagnostic test is recommended for PEI, anthropometric data such as Body Mass Index, serial assessment of clinical symptoms, and nutritional markers such as the levels of fat-soluble vitamins, plasma proteins, minerals, and serum lipids may also be considered in the diagnostic schema of PEI [72,73]. 

Overall, no single test or screening tool is recommended for PEI. Whereas CFA remains the gold standard of PEI, it is invasive and cumbersome to perform, and its alternative, the ^13^C-mixed triglyceride breath test, is not widely available. A general diagnostic approach to PEI was developed by the Australasian Pancreatic Club (APC), utilizing clinical symptoms, pre-test probability of PEI, and FE-1 measurement [54]. Patients with a high likelihood of PEI, such as those who have undergone total pancreatectomy, or have tumors that destroy the head of the pancreas are recommended a trial of pancreatic enzyme replacement therapy (PERT), while other patients are recommended to undergo further imaging or assessment of pancreatic function [54]. This is supported by a 2020 review from Pezzilli et al. [74], which suggested that the prevalence of PEI in patients with pancreatic head tumors is high enough that they should be treated with PERT without investigation. Similarly, the United Kingdom practical guidelines recommend an initial measurement of FE-1 when there is suspicion of PEI [73]. Based on these guidelines, a suggested approach to the diagnosis of PEI in pancreatic cancer is summarized in Figure 1B. 

## 4. Prevalence of PEI in Pancreatic Cancer

The prevalence of PEI in pancreatic cancer is still not well researched and, with a lack of awareness and/or variation in diagnostic testing, has been difficult to diagnose and treat. In a 2020 systematic review and meta-analysis that included 11 studies, PEI was estimated to affect 72% of patients with advanced pancreatic cancer [13]. Interestingly, while patients with pancreatic head tumors were more likely to suffer from PEI, 32% of patients with pancreatic body or tail tumors were also determined to have PEI [13]. Surgical resection is an independent risk factor of PEI as well. For example, Roeyen et al. [75] performed a study of 78 patients undergoing pancreaticoduodenectomy for oncological indications, finding that 20.5% of patients had PEI preoperatively while 64.1% of patients required PERT postoperatively. The rate of PEI after surgical resection can be impacted by the type of surgery performed. By definition, 100% of patients undergoing total pancreatectomy will have PEI. Reported postoperative rates of PEI after pancreaticoduodenectomy (PD) and distal pancreatectomy (DP) vary, although PD is generally associated with a higher risk of PEI [76,77,78]. In a 2016 systematic review, Tseng et al. [77] reported the median preoperative and postoperative prevalence of PEI for patients undergoing PD to be 44% and 74% (36–100%), respectively. Patients undergoing DP had a median preoperative prevalence of 20% and a postoperative prevalence of 67–80% [77]. Additionally, the use of pancreaticojejunal anastomosis rather than pancreaticogastric anastomosis following pancreaticoduodenectomy has been demonstrated to help preserve pancreatic exocrine function [79]. For patients undergoing resection, remnant pancreatic volume is also a predictive factor for postoperative PEI [80]. In a recent systematic review and meta-analysis of 20 studies, Budipramana et al. [81] further identified preoperative main pancreatic duct diameter >3 mm, hard pancreatic texture, and the use of adjuvant chemotherapy as risk factors for the development of pancreatic insufficiency. 

The prevalence of PEI among patients with locally advanced and metastatic PDAC is more difficult to determine accurately and would be better informed by further research. This may be an underappreciated problem among this subset of patients (which represents most patients with PDAC). Diagnostic challenges in this setting might stem from a lack of knowledge of PEI and the overwhelming symptomatic nature of PDAC diagnosis, which can be associated with symptoms of abdominal pain, discomfort, nausea/ vomiting, and bowel disturbances, among others. Many of these symptoms overlap with the symptoms of PEI. Likewise, systemic chemotherapy regimens commonly prescribed in this setting (e.g., FOLFIRINOX or gemcitabine/nab-paclitaxel) might be associated with considerable digestive toxicities which might further compound the accuracy of diagnostic reasoning. Moreover, patients and providers alike become rightly occupied by questions of prognosis, survival, and systemic treatment options for this lethal disease. Therefore, it is possible that PEI takes a back seat in this setting and is never properly addressed among many patients, which is unfortunate as the treatment of PEI can greatly impact their quality of life. Overall, specialized nutrition support from a dietitian is strongly recommended for all PDAC patients [82]. 

## 5. Practical Considerations of PEI Management

The optimal management of PEI in pancreatic cancer has been previously outlined in multiple international consensus statements, as summarized in Figure 1B [54,59,73,83]. General principles of PEI management involve a standardized approach to PEI diagnosis, dietary consultation, and the use of PERT. A comprehensive nutritional assessment, intake of smaller and more frequent meals, as well as replacement of any deficiencies in fat-soluble vitamins, vitamin B12, iron, and lipoproteins, are also important aspects of PEI management [54]. Here, we discuss some important practical considerations in the prescription of PERT for PEI. 

The goal of PERT is the delivery of pancreatic enzymes such as lipase, amylase, and protease into the intestinal lumen with meals to allow for proper digestion and absorption [54,59]. Most available pancreatic enzyme formulations are encapsulated into microgranules or minimicrospheres that dissolve at pH > 5.5 or higher to prevent the inactivation of lipase in the acidic environment of the stomach. Although uncoated formulations are also available [59,83,84], they are not indicated in patients with gastric secretions. The use of coated PERT has been associated with greater weight gain [85]. In addition, a 2017 meta-analysis also demonstrated a trend toward improved CFA when coated enzymes were compared to uncoated enzymes [86]. Uncoated PERT may theoretically be used for patients who have undergone gastrectomy [85] but are otherwise not recommended for PDAC patients.

Both uncoated and coated PERT have been used in the management of pain associated with chronic pancreatitis, although the use of uncoated PERT in PDAC associated with PEI is more limited [83,87]. This further supports the common practice of using coated PERT in pancreatic cancer patients. Multiple preparations of encapsulated pancreatic enzymes are commercially available, which have demonstrated variation in the distribution of particles, lipase activity, and release of enzymes at acidic pH levels in the in vitro setting [88,89]. Nevertheless, no direct comparison of efficacy has been made for pancreatic cancer patients. 

Proton pump inhibitors (PPIs) are commonly prescribed medications for the suppression of gastric acid and the treatment of acid-related disorders. Interestingly, there have been multiple epidemiological studies linking PPIs to an increased risk of pancreatic cancer and other gastrointestinal malignancies [90,91]. The impact of PPIs on PDAC outcomes is controversial, although recent experimental evidence has suggested a protective role [92,93]. In the setting of PEI, PPIs may act to further protect pancreatic enzyme replacements from degradation in the acidic environment of the stomach [83]. Nevertheless, routine use of PPIs concomitantly with PERT is not recommended. PPIs may be considered for patients who do not have an adequate response to PERT, or who have symptoms of dyspepsia [74,83,94,95,96]. 

Certain oral anticancer medications that are used in the treatment of PDAC (e.g., Capecitabine) may have reduced systemic exposure due to the high pH of the stomach. Capecitabine efficacy has been questioned among patients with colorectal cancer and gastroesophageal cancer when PPIs were used concurrently [97,98]. It is not entirely clear if raising the pH using PPIs would compromise the efficacy of Capecitabine or not. It is also not clear if alternative acid suppressive strategies would improve the absorption of Capecitabine and/or compromise the efficacy of PERT in this setting. Further research is necessary to define the impact of long-term PPI use on the efficacy of Capecitabine and other chemotherapeutic agents in the context of PDAC.

One recommended starting dose of PERT is approximately 40,000–50,000 units of lipase with meals and 25,000 units of lipase with snacks, to be titrated to the relief of clinical symptoms [59,83]. An alternative dosing strategy is 500–2500 units of lipase/kg/meal, half for snacks, to a maximum of 10,000 units of lipase per kg per day [99]. A sensible approach in clinical settings would be to titrate up to the initially recommended dosing over the course of several days. Regular assessment of nutritional status and symptoms is crucial for patients with PEI. For patients who do not respond to PERT, it is also important to ensure that patients understand the importance of proper use and compliance. This is underscored in a 2019 study by Barkin et al. [100], which demonstrated that only 66% of patients appropriately prescribed PERT were compliant and took PERT appropriately. Importantly, patients should be recommended to take PERT with meals and snacks, not after meals or scheduled throughout the day [73,100]. Dosage increases and the addition of PPIs should be considered if patients do not have relief of symptoms (Figure 1B). While there is some variation between different guidelines as to whether a specific maximum dose for PERT exists, it is important to exclude alternate causes for clinical symptoms when dosages exceed 100,000 units of lipase with meals without relief of symptoms [54,73]. Patients should also be counseled that the symptoms of PEI may not resolve completely, even with the appropriate use of PERT [74]. 

## 6. Appropriate Use of PERT for Pancreatic Cancer

Several international studies have examined the proportion of pancreatic cancer patients with PEI, in both resectable and non-resectable disease, that are prescribed appropriate PERT (Table 1). In a retrospective study of 129 patients with metastatic pancreatic cancer that were referred to palliative care, Landers et al. [101] found that while 72% of patients had symptoms suggestive of pancreatic insufficiency, only 21% of patients were prescribed PERT. The authors identified several possible contributing factors, including a lack of awareness of PEI or the benefits of PERT and insufficient screening initiatives [101]. Similarly, McCallum et al. [102] studied 183 patients with diagnoses of PDAC and pancreatic neuroendocrine tumors (PNET). In total, 63% of patients had symptoms to suggest PEI, although only 43% of patients received nutritional interventions such as PERT. Interestingly, patients who had received nutritional interventions were more likely to receive systemic chemotherapy, potentially because the nutritional interventions improved patients’ functional status to a point where they were deemed appropriate for chemotherapy [102]. The authors also identified several barriers to appropriate PERT, including clinicians’ assumptions that pancreatic cancer patients will have weight loss, lack of training in screening for PEI, and the burdensome testing for PEI. 

The attitudes of clinicians toward PERT were formally evaluated in a study that surveyed 208 hepato-pancreato-biliary surgeons [103]. Whereas the majority believed that PERT improves nutritional status, only a minority believed that PERT improves overall survival (19.7%), disease-free survival (6.25%), or the rate of postoperative complications (28.8%) [103]. Furthermore, inconsistencies were identified in how patients were selected for PERT, the duration of therapy, and how treatment efficacy was monitored [103]. Postoperative PEI can commonly manifest up to 3 months after surgery [104], which can contribute to the underdiagnosis of PEI if symptoms are not assessed at follow-up appointments. Interestingly, regular interaction with dietician colleagues has also been shown to improve the recognition of malnutrition by clinicians [105]. Additionally, a United Kingdom (UK) study of patients with pancreatic cancer or malignant biliary obstruction demonstrated that the rate of PERT was higher for patients with resectable disease when compared to those with unresectable disease (45.3% compared to 74.4%, *p* < 0.001) [106]. PERT prescription was increased for patients who had a consult with a dietitian and those who had been managed at specialized hepatopancreaticobiliary or pancreatic units [106]. Unfortunately, there is some evidence of an inequality of care where patients being treated with palliative intent are less likely to receive PERT, perhaps due to a lack of perceived benefit [106]. 

The patient perspective of PEI provides additional clues regarding its sub-optimal management. Dunleavy et al. [107] performed a qualitative study that identified gaps in patient education around PERT, to the point where several patients attributed symptoms of malabsorption to PERT itself. In addition, the social implications of needing to take PERT with meals and concerns around side effects and the frequency of dosing may contribute significantly to inconsistent adherence to treatment [107]. Lander et al. [108] demonstrated that a structured consultation with a specialized palliative team, regular dietary reassessment, and enhanced health literacy allowed patients to become more involved in managing PERT, thus improving adherence.

Overall, these studies demonstrate the underdiagnosis and undertreatment of PEI by clinicians, likely resulting from a combination of factors including challenging diagnostic tests for PEI, lack of training, and a lack of perceived benefit to long-term outcomes such as overall survival, especially for patients with unresectable disease. Longitudinal nutritional support, education, and peer support may serve to improve patient understanding of PEI as well as treatment adherence. 

**Table 1:** Summary of studies examining the frequency of appropriate treatment for exocrine pancreatic insufficiency in patients with pancreatic cancer.

Reference	Study Population	Resectable/Unresectable	Study Location	Findings
[101]	Retrospective study of 129 patients with metastatic pancreatic cancer	Both	New Zealand	Whereas 70% of patients had symptoms of malabsorption, only 21% were prescribed PERT.
[102]	Retrospective study of 183 patients with PDAC and pNETs	Both	UK	63% of patients had symptoms of pancreatic enzyme insufficiency, and 43% received a nutritional intervention (PERT, nutritional supplementation, or dietician referral).
[100]	262 patients diagnosed with pancreatic cancer	Both	USA	85% of patients surveyed discussed PERT with their healthcare provider, and 75% were prescribed PERT. Of the patients prescribed PERT, only 65% were prescribed appropriately (with all meals and snacks).
[109]	32,461 individuals identified with pancreatic cancer from US insurance claims	Both	USA	1.9% of patients with pancreatic cancer were tested for exocrine insufficiency, and 21.9% filled a prescription for PERT.
[106]	1350 individuals with malignant pancreatic, peri-ampullary lesions or malignant biliary obstruction	Both	UK	74.4% of patients with resectable disease and 45.3% of patients with unresectable disease were prescribed PERT. The rate of PERT prescription was increased for patients who had dietician referral (*p* = 0.001), management at hepatopancreaticobiliary (*p* = 0.049), or pancreatic unit (0.009). The rate of prescription was inversely correlated with the Charlson comorbidity score.
[103]	Survey of 208 hepato-pancreato-biliary surgeons	N/A	USA	86.5% of surgeons prescribed PERT for at least some patients with resectable or borderline resectable PDAC, and 28.1% ordered tests to confirm exocrine pancreatic enzyme insufficiency before starting PERT. 19.7% of surgeons believe PERT impacts overall survival, and 6.25% of surgeons believe PERT impacts disease-free survival.
[110]	95 patients who underwent pancreaticoduodenectomy for suspected/confirmed pancreatic or periampullary (pre)malignancy	Resectable	The Netherlands	97% of patients developed pancreatic exocrine insufficiency, and 48–79% of patients had vitamin D or K deficiencies. 0–50% of patients with deficiencies received vitamin supplementation.

## 7. Impact of PERT on Patient Outcomes

Several studies have demonstrated a positive impact of PERT on the nutritional status of patients with pancreatic cancer, although the majority have been retrospective (Table 2). Interestingly, pre-clinical models have also demonstrated that PEI drives peripheral tissue wasting [111]. Results from randomized controlled trials have been generally limited to small cohorts of patients and have been challenging to interpret due to conflicting results. Bruno et al. [112] first performed a randomized trial of 21 patients with unresectable pancreatic head tumors with suspected pancreatic duct obstruction, demonstrating that PERT allowed patients to gain weight while improving fat absorption and daily energy intake. These results are unfortunately not supported by later trials [113,114,115]. In the largest trial to date, Saito et al. [115] examined 88 patients with unresectable pancreatic cancer that were randomized to Pancrelipase and control groups. When comparing the two groups, the authors did not find a statistically significant difference in BMI at 8 weeks or other nutritional markers. The authors hypothesized that the absence of appropriate dietary consultation limited the degree of oral intake for patients and that intensification of systemic chemotherapy with its associated gastrointestinal toxicities limited the impact of PERT in comparison to the earlier study [112,115]. Beyond objective measures of nutrition, symptoms of PEI can also have a significantly negative impact on patients’ quality of life if not properly treated [116]. 

In addition to its effects on nutrition and quality of life, the potential impact of PERT on survival outcomes in pancreatic cancer has also been evaluated. Interestingly, a study by McCallum et al. [102] that included patients with both PDAC and pancreatic neuroendocrine tumors (PNETs) demonstrated that nutritional interventions for PEI have the potential of improving not only functional status but also eligibility for systemic chemotherapy. However, none of the three randomized controlled trials that specifically examined the impact of PERT on overall survival have demonstrated a statistically significant improvement in overall survival [113,114,115]. Of note, Zdenkowski et al. [114] did identify a numerically greater median survival in the PERT group, although this did not reach statistical significance, owing to the limited sample size (67.6 weeks in the pancreatic extract group compared to 17 weeks in the control group, n = 18). An independent systematic review that only included four randomized controlled trials again failed to identify a significant impact of PERT on overall survival, although the authors acknowledged small sample sizes, heterogeneity in trial design, and endpoints as limitations [117]. 

There have been multiple retrospective analyses of larger cohorts of patients that have demonstrated improved survival outcomes [102,118,119,120]. In the most recent and largest retrospective study to date, Roberts et al. [120] studied 807 patients with pancreatic adenocarcinoma (PDAC) and demonstrated a longer median survival in patients receiving PERT (274 vs. 140 days, *p* < 0.001). Importantly, the survival benefit of PERT was maintained even in the subgroup of PDAC patients who did not undergo surgical resection or palliative chemotherapy, representing a significant proportion of patients with advanced pancreatic cancer [120]. In a recent systematic review and meta-analysis that included 11 studies, PERT was associated with an overall survival benefit of 3.8 months (12.6 vs. 8.7 months, *p* = 0.002) [13]. 

The conflicting evidence for the survival impact of PERT in pancreatic cancer is limited by the small sample sizes of currently available randomized controlled trials, heterogeneity in patient populations (e.g., the proportion of patients with pancreatic head tumors), and availability of specialized dietary support for these patients. Most studies that investigate the impact of PERT have also focused on patients with unresectable disease (Table 2).

Further studies, ideally multi-center, randomized controlled trials, are necessary to assess the impact of PERT on patient outcomes and may serve to increase the awareness of PEI among clinicians. Alternatively, advocacy initiatives to incorporate PERT as part of the best practices for PDAC management would also provide supporting evidence. One such trial is the Dutch Pancreatic Cancer Project (PACAP), which assesses the impact of best practices in palliative chemotherapy, PERT, and metal biliary stents on overall survival and patient quality of life [121]. Despite the limitations of current literature, there is certainly an under-recognition of PEI in pancreatic cancer patients and of the potential impact of PERT on the quality of life for patients with PDAC.

**Table 2:** Summary of studies examining the impact of pancreatic enzyme replacement (PERT) on patient outcomes for pancreatic cancer.

Reference	Study Population	Resectable/Unresectable	Intervention	Findings
[122]	Prospective study of 12 patients with biopsy-proven pancreatic ductal adenocarcinoma	Unresectable	Pancreatic Tablets	Pancreatic enzyme supplementation improved absorption for patients with moderate to severe fat or protein malabsorption but not in patients with mild fat or protein malabsorption.
[112]	Randomized controlled trial of 21 patients with unresectable pancreatic cancer with suspected biliary obstruction	Unresectable	Panzytrat 25,000 (coated)	The use of pancreatic PERT allowed for an average weight gain of 1.2% (0.7 kg), while placebo control patients lost 3.7% (2.2 kg). Patients on PERT also had increased fat absorption coefficient and total daily energy intake compared to placebo controls.
[113]	Randomized controlled trial of 67 patients with unresectable pancreatic cancer (34% pancreatic head tumours)	Unresectable	Norzyme (coated)	No statistically significant difference in body weight, patient-generated subjective global assessment score, quality of life, or overall survival was observed between the two groups.
[102]	Retrospective study of 183 patients with PDAC and pNETs	Both	Nutritional intervention (including PERT)	Patients who received PERT were more likely to receive systemic chemotherapy compared to patients who did not receive PERT (65.8% vs. 50%, univariate *p*-value 0.03).
[118]	Retrospective analysis of 469 patients undergoing pancreaticoduodenectomy for PDAC, cholangiocarcinoma, ampullary carcinoma, or duodenal carcinoma	Resectable	PERT	43.1% of patients received PERT. PERT use was independently associated with improved survival (HR 0.72, 95% CI 0.52–0.99, *p* = 0.044). The effect of PERT on patient survival was mostly observed in patients with a pancreatic duct ≥3 mm.
[119]	Prospective observational study of 46 patients with pancreatic cancer on PERT and 45 historical controls	Unresectable	Pancrelipase	Patients on PERT maintained nutritional markers in comparison to historical controls (BMI unchanged at 16 weeks for PERT, decreased from 21.7 to 21.2, *p* < 0.001 for controls; serum albumin + 0.1 mg/dL for PERT, −0.1 mg/dL in historical controls although not statistically significant). The use of PERT was associated with significantly higher overall survival on univariate analysis, although not significant after adjusting for other factors (HR 0.73; *p* = 0.367).
[114]	Randomized controlled trial of 18 patients with advanced pancreatic cancer	Unresectable	Creon 25,000 (coated)	Pancreatic extract supplementation was not associated with a significant difference in BMI or quality of life. Median overall survival was numerically greater in the pancreatic extract group compared to the control group (67.6 weeks compared to 17 weeks), although did not reach statistical significance.
[115]	Randomized controlled trial of 88 patients with unresectable pancreatic cancer receiving chemotherapy	Unresectable	Pancrelipase	The addition of PERT did not significantly improve BMI, other nutritional markers, or median overall survival.
[123]	Retrospective analysis of 160 patients with unresectable pancreatic cancer	Unresectable	Creon 25,000 (coated)	The use of PERT was significantly associated with improved survival (HR 1.81, 95% CI 1.23–2.66, *p* = 0.002 in multivariate analysis). Survival of patients treated with PERT due to symptoms of pancreatic insufficiency was similar to that of patients who did not receive PERT due to the absence of symptoms (HR 1.12, 95% CI 0.67–1.89).
[124]	Prospective study of 29 patients with metastatic pancreatic adenocarcinoma, excluding those with ECOG ≤ 3 or PPS < 60%	Unresectable	Creon 25,000 (coated)	The use of PERT was associated with a statistically significant improvement in symptoms (diarrhea, pancreatic and hepatic pain, bloating/gas).
[120]	Retrospective observational cohort study of 807 patients with pancreatic adenocarcinoma on PERT matched to non-PERT treated controls	Both	PERT	Patients receiving PERT had longer median survival than patients not receiving PERT (274 vs. 140 days, *p* < 0.001). The effect was preserved in subgroup analysis, including in patients not receiving chemotherapy or surgery.

## 8. Conclusions

PEI is a common complication of pancreatic ductal adenocarcinoma, with implications on patients’ nutritional status, quality of life, and overall prognosis. Existing guidelines recommend a standardized nutritional assessment of PDAC patients, the use of FE-1 as an adjunctive test, rapid initiation of PERT for patients that are at high risk of PEI, and regular nutritional assessments. Unfortunately, PEI remains an underrecognized and undertreated condition due to barriers such as a lack of training among clinicians, variation in perceptions regarding its importance in patient outcomes, and uncertainties in the diagnostic approach. Importantly, patients who suffer from PEI benefit from education surrounding this condition and a multidisciplinary approach to its management. Future multi-center randomized trials are necessary to clarify the current uncertainty about the impact of PEI on patient outcomes.
